# Psychological Symptoms in Parents Who Experience Child-to-Parent Violence: The Role of Self-Efficacy Beliefs

**DOI:** 10.3390/healthcare11212894

**Published:** 2023-11-03

**Authors:** Aitor Jiménez-Granado, Liria Fernández-González, Joana del Hoyo-Bilbao, Esther Calvete

**Affiliations:** Department of Psychology, Health Sciences Faculty, University of Deusto, 48007 Bilbao, Spain; aitor.jimenez@deusto.es (A.J.-G.); joana.delhoyo@deusto.es (J.d.H.-B.); esther.calvete@deusto.es (E.C.)

**Keywords:** child-to-parent violence, parents, impact, psychological symptoms, self-efficacy beliefs

## Abstract

Previous research suggests that parents involved in child-to-parent violence (CPV) experience shame, judgment, and a lack of social support, often accompanied by feelings of self-blame and helplessness as well as a deterioration in their perception of self-efficacy and their parenting skills. All of these factors may impact parents’ mental health. However, there is a research gap concerning the consequences of CPV among parents. Therefore, the objective of this study was to examine the relationship between CPV and psychological symptoms (i.e., depression, anxiety, hostility, obsessive–compulsive, interpersonal sensitivity, and somatization) in parents based on their perceptions of self-efficacy. The sample was composed of 354 participants: 177 parents (83.1% mothers) and their 177 children (53.4% boys; *M*_age_ = 13.27). CPV was reported by both parents and their children. In addition, parents reported their self-efficacy beliefs and psychological symptoms. The results showed that CPV was negatively associated with parents’ psychological symptomatology, except for somatization. Moreover, self-efficacy beliefs explain part of the indirect association between CPV behaviors and psychological symptoms in parents. Overall, our findings provide evidence for the potential impact of CPV on mental health in parents and suggest the relevance of reinforcing their self-efficacy beliefs.

## 1. Introduction

Child-to-parent violence (CPV) is an increasing social problem in which researchers have turned their attention, mainly over the last decade (for a comprehensive review, see [1]). Different terms (e.g., child-to-parent aggression, parent abuse, or youth-to-parent aggression) and definitions have also been proposed [2]. Classical definitions highlighted the intention of children to gain power over parents [3] or the feeling of threat or intimidation among parents [4]. Recently, a group of experts from the Spanish Society for the Study of Child-to-Parent Violence (SEVIFIP) avoided including the motivation of the behaviors in the definition, as they can be instrumental (in order to obtain some benefits) as well as reactive (motivated by anger and/or emotional distress), or defensive (defending oneself or someone else) [5]. According to these experts, CPV should be defined as repeated physical, psychological, or economic violence directed at parents or those who take their place [6]. Physical aggressions are those in which direct physical harm (such as pushing or kicking) or indirect physical harm (using an object) is inflicted on parents. Psychological aggressions include behaviors (verbal or non-verbal) such as insulting or humiliating their parents. Finally, economic violence refers to theft or financial abuse [1].

The differences in conceptualizations and nomenclature and measurement and sampling have hindered the proper study of CPV and consensus on its prevalence [1,7,8]. For example, a recent community study in Germany found that 42.8% of the assessed adolescents reported psychological aggressions at least once in the last year, and 4.6% of them reported physical aggressions [9]. Another study in Chile in the same year found that 86.1% of the children assessed in a community sample reported at least one type of CPV behavior in the last year, with 82.2% reporting psychological aggressions and 20% physical aggressions [10]. In Spain, another recent community study conducted during the COVID-19 pandemic reported that 60.3% of the children engaged in at least one type of CPV behavior, with insulting being the most prevalent behavior [5]. Most studies have found that mothers are the main target of these behaviors [1,11]. However, other studies found no differences between fathers and mothers as targets [5]. Moreover, the majority of studies have been based on adolescents’ reports, while parents’ reports have usually been neglected (for exceptions, see [12,13,14]). When parents’ reports have also been considered, it has been found that parents and their children tend to differ in their reports, with children reporting a higher number of aggressive behaviors than parents [12].

Despite the social relevance of the phenomenon and the high prevalence rates found in community samples, the effect of CPV on parents’ psychological symptoms has been understudied [1,15,16]. The negative impact of CPV on parents may exacerbate the difficulties parents encounter when they try to put an end to it [17]. Therefore, increasing our knowledge about the consequences of CPV on parents would facilitate a deeper understanding of the phenomenon, as well as improve prevention and intervention strategies.

### 1.1. CPV and Psychological Symptoms in Parents

The relationship between victimization and psychological symptoms has been well established in relation to other forms of family violence. For example, child maltreatment has been found to predict the development of depression, anxiety, and PTSD symptoms in children (see [18], for an extensive review and meta-analysis). Similarly, reviews on intimate partner violence have shown that depressive and PTSD symptoms are highly prevalent among victims (see [19,20]). Nevertheless, while the impact of these other types of family violence on psychological well-being has been widely studied, there is a gap in research regarding the impact of CPV behaviors on parents [1].

Studies on parents of children with externalizing problems provide valuable information that could be extrapolated to this field. For instance, the general externalizing problems of children predict parental distress [21,22], depression [22,23], and anxiety [24]. Moreover, some qualitative studies suggest that parents involved in CPV experience high levels of psychological difficulties (e.g., [25,26]). In one study, mothers and grandmothers immersed in CPV described their experience as “an emotional rollercoaster” between “unconditional love” and “hate” [26]. Additionally, in a recent scoping review, parents described feelings of self-blaming, impotence, insecurity, social isolation, and a lack of support [27]. Thus, the available research suggests that CPV may have a negative impact on the mental health of parents [28].

In addition to the limited research on the impact of CPV on parental mental health, there is another gap related to the cognitive mechanisms through which this impact occurs. In this regard, the theory of the mutually coercive cycle regarding CPV states that parents and children fall into a mutual influence dynamic, where parental authority could be diminished [29]. This loss of authority may be accompanied by a decline in self-efficacy beliefs [27], which could explain the negative impact of CPV on parental mental health, as elaborated below.

### 1.2. Victimization and Self-Efficacy Beliefs in Parents

Previous studies have identified cognitive mechanisms that may contribute to explaining the impact of different types of victimization on psychological symptoms, such as negative self-concept [30] and maladaptive schemas [31,32]. Self-efficacy may also be impaired by victimization, including peer victimization [33], sexual victimization [34], and intimate partner victimization [35], contributing to the development of psychological symptoms.

Self-efficacy refers to the belief of being able to influence the environment through one’s actions and means in order to achieve different outcomes [36]. This belief changes over time and is influenced by personal skills, such as parental communication skills [37], and relational variables, such as marital/couple functioning [38] and children’s internalizing and externalizing behaviors [39,40].

Self-efficacy beliefs are important for parents’ development of adequate parenting skills in order to enforce appropriate discipline [37,41] as well as for supporting their psychological adjustment when facing difficulties (for a review, see [42]). In this vein, a study found that self-efficacy beliefs were negatively related to depression symptoms in caregivers of children with oppositional defiant disorder [43]. Further, parents’ self-efficacy beliefs have been shown to mediate the relationship between children’s behavioral problems and parental depression and anxiety symptoms [44].

### 1.3. The Present Study

There is a gap in the literature regarding the impact of CPV on parents. However, the few qualitative studies that have been conducted on this topic suggest that parents who experience CPV suffer from a wide range of consequences that could lead to mental health issues [26]. In this sense, parents who experience CPV reported depression and anxiety symptoms [27], and these symptoms are the most frequently evidenced in other types of victims [20]. Additionally, both somatization [45,46] and obsessive–compulsive symptoms (such as ruminative thoughts, for example) [47] are symptoms highly linked to stressful situations. Moreover, hostility and interpersonal sensitivity are reported in victims of CPV [26,27].

Moreover, a recent review of explanatory factors suggested that parents involved in CPV situations tend to have a poor perception of their self-efficacy as parents [27]. Although the cognitive mechanisms behind the impact of CPV on parental mental health have not been studied, findings related to other types of victimization suggest the potential mediating role of self-efficacy beliefs.

Accordingly, this study aimed to examine whether CPV (evaluated based on reports from parents and their children) relates to parents’ psychological symptoms (i.e., depression, anxiety, hostility, obsessive–compulsive, interpersonal sensitivity, and somatization) and whether self-efficacy beliefs contribute to explaining this association. Considering previous empirical evidence, the following hypotheses were proposed: (1) CPV will be associated with lower self-efficacy beliefs [27] and higher levels of psychological symptoms [25,26,28], and (2) self-efficacy will explain part of the association between CPV behaviors and psychological symptoms [44].

## 2. Materials and Methods

### 2.1. Procedure

After the Ethics Committee of the University of Deusto approved the study, the principals of 20 high schools from Bizkaia (Spain) were contacted to explain the study’s objectives and request collaboration. Five high schools agreed to take part in the study. Parents received information regarding their own and their children’s participation in the form of a passive consent form sent online by the school. Adolescents were provided with information documents through the schools and also received explanations in person from the researchers on the day of assessment. Both children and parents were informed that their participation was voluntary and anonymous (a code was used to link children’s and parents’ answers) and that they could withdraw their permission at any time, without consequences.

This study is part of a larger project in which 905 adolescents participated. For this study, we selected those adolescents for whom at least one parental figure had completed the study questionnaires. In this sense, the final sample consisted of 177 dyads (19.56% of the total sample). Responses were collected between December 2020 and September 2021. Adolescents answered the questionnaires online through Qualtrics^®^, in their classrooms, and in the researcher’s presence to clarify any questions. Parents answered the questionnaires online through Qualtrics^®^ at home.

### 2.2. Participants

The sample consisted of 354 participants: 177 parents (83.1% mothers and 16.9% fathers) and their 177 children (53.4% boys, 43.6% girls, and 3.1% non-binary; *M*_age_ = 13.27, *SD* = 1.14). In terms of nationality, 95.5% of the participants were Spanish, 4.0% were foreigners (mainly South and Central Americans), and 0.6% did not report their nationality. Regarding family structure, the parents reported that 44.1% of the children lived only with the mother, 42.4% lived with both parents, 8.5% lived with the father, and 2.8% lived with the mother and a new partner of the mother or other adult figure (grandfather, grandmother, or both), and 1.7% lived with both parents in any shared custody arrangement. The parents’ reported income per family unit was as follows: EUR <12.000 (6.2%), between EUR 12.000–18.000 (14.7%), EUR 18.000–24.000 (14.7%), EUR 24.000–36.000 (22.6%), EUR 36.000–50.000 (28.2%), and EUR >50.000 (13.6%).

### 2.3. Measures

Child-to-Parent Violence: The Revised Child-to-Parent Aggression Questionnaire (CPAQ-R; [5]) was used to assess child-to-parent violence reported by parental figures and their children. While this questionnaire was developed to be answered by children, it can be also adapted to be answered by the parents. The original version of the CPAQ [48] was validated for both adolescents and parents [49], indicating the acceptability of adapting the new version for parents as well. The questionnaire consists of 9 items that ask about the frequency of CPV behaviors (e.g., “You made fun of him/her” (children) or “He/she made fun of you” (parents)) in the last three months, with answers ranging from 0 (never) to 3 (it has happened six or more times). Cronbach’s alphas for this study sample were 0.78 for parents’ reports and 0.85 for children’s reports.

Psychological Symptomatology: The Spanish version [50] of the Symptoms Assessment-45 Questionnaire (SA-45; [51]) was employed to assess the parents’ psychological symptoms. The SA-45 is a short form of the Symptom Checklist-90 [52], a widely used scale for the assessment of psychopathological symptoms in clinical or community adults. It consists of 9 subscales (5 items each), with answers ranging from 0 (not at all) to 4 (very much). In this study, we used the depression, anxiety, hostility, obsessive–compulsive, interpersonal sensitivity, and somatization sub-scales. Cronbach’s alphas for this study sample were 0.80 for depression, 0.79 for hostility, 0.82 for anxiety, 0.77 for obsessive–compulsive, 0.83 for interpersonal sensitivity, and 0.86 for somatization, consistent with other studies [53].

Self-efficacy Beliefs: Parents’ self-efficacy beliefs were assessed using the General Self-efficacy Scale [54] (Spanish version by [55]). The scale is made up of 10 items (e.g., “If I find myself in a difficult situation, I generally find a solution”) that are answered on a 4-point scale ranging from 1 (totally disagree) to 4 (totally agree). The General Self-efficacy Scale consists of a unique general factor and is widely used in assessing personal self-efficacy beliefs in different situations, demonstrating adequate psychometric properties [56]. In this study, Cronbach’s alpha was 0.87.

### 2.4. Data Analysis

As the data did not follow a normal distribution (see asymmetry and skewness analyses in Table 1), descriptive statistics and Spearman’s Rho correlations were calculated using IBM^®^ SPSS^®^ Statistics 28. To calculate CPV percentages, the participants were classified as aggressive if they reported at least one act of aggression in any of the behaviors described. A Chi-square analysis was conducted to examine the congruence between parents’ and children’s reports, and the effect size was calculated based on Cramer’s V.

To test the hypotheses, Structural Equation Modeling with observed and latent variables was performed with MPLUS 8.8 [57], using the Robust Maximum Likelihood (MLR) estimation method. The models’ goodness of fit was evaluated using the Chi-square test (χ^2^), Comparative Fit Index (CFI), Non-normed Fit Index (NNFI), Root Mean Square of Error Approximation (RMSEA), and Standardized Root Mean Square Residual (SRMR). According to [58], CFI and NNFI values of 0.95 or higher and RMSEA and SRMR values lower than 0.08 indicate an adequate fit.

Considering previous findings, which showed incongruencies and different CPV prevalence rates between parents’ and children’s reports [12], the CPV variable was modeled as a latent variable that included indicators from both parents’ and their children’s reports. In particular, the latent CPV variable was modeled using six three-item parcels as indicators (three from parents’ reports and the corresponding three parallel parcels from children’s reports). The items were assigned to parcels by conducting an exploratory factor analysis, with all the CPAQ-R items answered by the parents. To control the residual variance in the items answered by children, a method factor was included in which the parcels of children’s reports were loaded [59,60].

The structural hypothetical model included paths from the latent CPV variable to self-efficacy beliefs and psychological symptoms, and paths from self-efficacy beliefs to psychological symptoms. Thus, we examined whether CPV was associated with psychological symptoms in the parents and whether this association was explained by self-efficacy beliefs. Additionally, in order to control the overlap between the outcomes, all the psychological symptoms (i.e., depression, anxiety, hostility, obsessive–compulsive, interpersonal sensitivity, and somatization) were allowed to covariate. To test the significance of the indirect effects of CPV on psychological symptoms through self-efficacy, a bootstrap analysis was conducted with 10,000 samples. The 95% confidence interval (CI) was analyzed (establishing a two-tailed distribution, thus using the percentile scores of 2.5 and 97.5), and it was considered to be significant if the results did not contain zero [61].

## 3. Results

Descriptive statistics (means, standard deviations, score ranges, asymmetry, and skewness) are described in Table 1.

Spearman’s Rho correlation coefficients are depicted in Table 2. The percentage of children and parents who reported at least one CPV behavior in the last three months was 52.6 and 51.9, respectively, χ^2^ (1) = 5, *p* = 0.006, V = 0.22. Regarding associations between variables, CPV reported by parents was negatively associated with self-efficacy beliefs and positively associated with symptomatology (Table 2). Self-efficacy beliefs were negatively and significantly associated with all the symptoms, with the exception of somatization. All the symptoms were positively and significantly associated with each other.

CPV reported by children was significantly associated with CPV reported by parents, although with a low effect size (*r* = 0.27). Moreover, the correlation coefficients of parents’ self-efficacy and symptoms with CPV were lower when CPV was reported by children than by parents. Specifically, only two correlation coefficients were significant: CPV (reported by children) with parents’ self-efficacy and hostility.

### Associations between CPV, Self-Efficacy, and Psychological Symptoms

**Figure 1:**
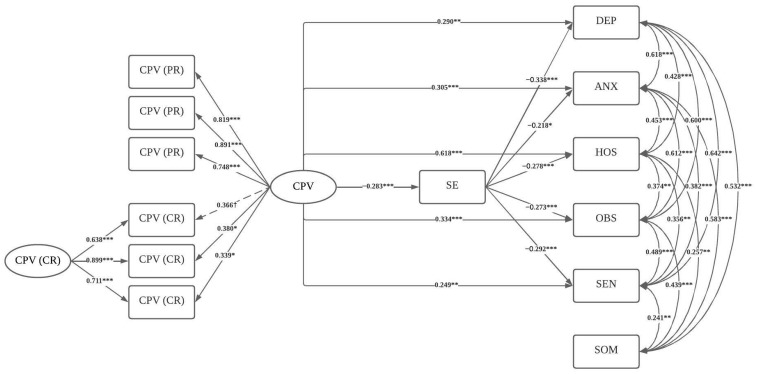
Structural Equation Model including the study variables. Note. *** *p* < 0.001, ** *p* < 0.01, * *p* < 0.05, † *p* < 0.10. CPV = child-to-parent violence; PR = parents’ report; CR = children’s report; SE = self-efficacy; DEP = depression; ANX = anxiety; HOS = hostility; OBS = obsessive–compulsive symptoms; SEN = interpersonal sensitivity; SOM = somatization. Standardized coefficients are shown in the figure. Slashed lines indicate marginally significant coefficients and straight lines indicate significant coefficients.

Figure 1 shows the model diagram with all the significant coefficients. The hypothesized model showed adequate fit indexes: MLR scaled—χ^2^ (41, *n* = 177) = 73.279; *p* < 0.001; RMSEA = 0.067; 90% CI [0.041, 0.091]; *p* (RMSEA <= 0.05) = 0.130; CFI = 0.973; TLI = 0.949; and SRMR = 0.049. The model explained 25.4% of the variance for depression, 17.8% for anxiety, 55.7% for hostility, 23.7% for obsessive–compulsive symptoms, 18.8% for interpersonal sensitivity, and 3.6% for somatization.

All the factor loadings of the CPV latent variable were significant, except for one of the indicators of the children’s report, which was marginally significant (*p* = 0.054). CPV was significantly associated with lower self-efficacy beliefs and with higher psychological symptoms, except for somatization. Self-efficacy beliefs were significantly associated with lower symptomatology, except for somatization. The covariances between symptomatology factors were all positive and statistically significant.

The bootstrapping procedure showed that the indirect associations between CPV and depression (95% CI: 0.031, 0.172), anxiety (95% CI: 0.008, 0.132), hostility (95% CI: 0.026, 0.130), obsessive–compulsive symptoms (95% CI: 0.023, 0.141), and interpersonal sensitivity (95% CI: 0.025, 0.149) through self-efficacy beliefs were statistically significant.

## 4. Discussion

This study sought to fill the gap in research on the impact of CPV on parents’ mental health. Specifically, the study examined the associations between CPV and psychological symptoms (i.e., depression, anxiety, hostility, obsessive–compulsive, interpersonal sensitivity, and somatization) through self-efficacy beliefs in parents. Consistent with our hypotheses, CPV was related to all psychological symptoms (i.e., depression, anxiety, hostility, obsessive–compulsive, and interpersonal sensitivity), except for somatization. In general, these findings are consistent with those obtained regarding victimization in other types of family violence (for reviews, see [18,19,20]) and the few previous qualitative studies on the impact of CPV on parents (for a scoping review, see [27]).

Specifically, in relation to anxiety and depression symptoms, a classic study found that parents involved in CPV had higher levels of both symptoms solely because they were conscious of the situation in which they were involved [62]. In addition, parents may struggle with daily life issues, along with feelings of self-blame, impotence, and insecurity [26,28,63], which are stressors that increase their vulnerability to anxiety and depression [64]. Moreover, in terms of obsessive–compulsive symptoms, parents tend to feel the need to be hypervigilant and keep their guard up at all times in their family environment [25]. With respect to interpersonal sensitivity, parents commonly report a feeling of being judged because of their parenting skills [63], which could cause them to perceive less social support and more social isolation [27], making them suspicious and reluctant to participate in social interactions. CPV has also been found to be related to higher levels of hostility in parents. Indeed, many parents feel angry when facing these behaviors and have reported emotional lability, sometimes in the form of hatred towards their children [26], as well as a loss of control [65].

Contrary to our expectations, somatization symptoms, although significantly and negatively associated with CPV in the bivariate analyses, were not related to CPV in the model that included all the variables. These results differ from previous studies that relate victimization with somatization [45,46]. However, other studies found that somatization was only related to control exerted by the victimizer and not to other victimization behaviors [66]. Controlling behavior was not assessed in this study because it is not specific to the phenomenon of CPV, so the lack of a relationship with somatization could be because of this fact.

The second hypothesis of the study was that self-efficacy beliefs would explain part of the association between CPV behaviors and psychological symptoms in parents. Our findings supported this hypothesis. CPV was associated with lower self-efficacy beliefs, and these beliefs in turn were associated with fewer psychological symptoms. This finding suggests that experiencing CPV may undermine parents’ perception of self-efficacy and contribute to the aforementioned psychological symptoms. Self-efficacy beliefs are dynamic and are influenced by external factors, such as children’s behavioral problems [39]. Thus, in the case of CPV, the presence of this type of violence could be affecting parents’ self-efficacy by undermining their confidence in their ability to manage the situation and causing them to feel diminished power in the relationship [3,27]. This reduction in confidence could partially explain the relationship between CPV and symptoms in parents, as reported in previous studies on parenting and children’s externalizing behaviors [44]. Even so, self-efficacy beliefs only explained part of the association between CPV and psychological symptoms, suggesting that other variables may be involved, such as feelings of shame or social isolation [27].

Additionally, the prevalence of CPV behaviors reported by children was slightly higher than the prevalence reported by their parents, and the congruence between the reports of children and their parents was small, which is consistent with previous studies [12]. Parents tend to report lower CPV rates, in comparison to their children, because they could be minimizing some of the behaviors of their children or even because they feel those behaviors as their own parental failure, as usually interpreted in society [12].

### Strengths, Limitations, and Future Directions

This is the first study to examine the associations between CPV, self-efficacy beliefs, and psychological symptomatology in parents, helping to fill a research gap regarding the impact of CPV on parents. Further, this is one of the few studies to assess CPV from the perspective of two groups of informants (i.e., parents and their children), ultimately forming 177 dyads. This overcomes one of the most frequently observed limitations in CPV studies, providing a more objective view of the phenomenon.

However, this study is not free of limitations, and the results should be interpreted with caution. First, the cross-sectional nature of the study prevents any conclusions regarding predictive relationships between the variables. Future longitudinal studies are needed to determine the directionality between variables as well as the mediating role of self-efficacy beliefs between CPV and parents’ symptomatology. Thus, alternative hypotheses cannot be ruled out, such as psychological symptoms reducing self-efficacy beliefs. Second, even though parental self-efficacy refers to a dimension of general self-efficacy, the scale used to assess self-efficacy beliefs in this study addressed general self-efficacy in different situations. Thus, future studies should focus on the particular dimension of parental self-efficacy in order to examine the study hypotheses. Third, the sample used a community sample, in which CPV behaviors and psychological symptoms were relatively low. The relations among the measured variables should be assessed in a clinical sample of parents of CPV offenders. In addition, the gender imbalance in the parents, with more mothers makes it impossible to determine the role of each of the parental figures in the analyzed relationships. This situation is consistent with the literature on parenting, which has shown that mothers are much more involved in parenting than fathers [67], and consistent with previous studies in CPV with parents as reporters [12], as stated. Therefore, future studies should try to involve more fathers and examine the relationships between fathers and mothers of children engaged in CPV behaviors.

## 5. Conclusions

Overall, the findings showed that CPV is related to psychological symptoms in parents. This relationship may be partially explained by a reduction in parents’ self-efficacy levels. Some implications for practice can be drawn from our findings. Specifically, the results highlight the importance of providing individual support for parents throughout the whole process, in addition to interventions targeting the children and the family as a whole, considering the effect that it may have on their mental health. Moreover, given that parents’ self-efficacy has an effect on their psychological well-being, it is important to help them regain a feeling of control over their family situations and develop effective strategies for managing harmful or complicated dynamics that may be affecting their psychological well-being.

## Figures and Tables

**Table 1:** Descriptive statistics of the study variables.

	Mean	SD	Range	Asymmetry	Skewness
1 CPV (CR)	0.18	0.31	0–3	4.03	24.14
2 CPV (PR)	0.16	0.26	0–3	3.23	15.25
3 Self-efficacy	2.96	0.41	0–3	−0.47	1.77
4 Depression	0.57	0.59	1–4	1.65	3.27
5 Anxiety	0.56	0.52	0–4	1.38	2.29
6 Hostility	0.29	0.29	0–4	2.59	7.84
7 Obsessive–compulsive	0.60	0.60	0–4	1.38	2.44
8 Interpersonal sensitivity	0.48	0.48	0–4	1.90	4.73
9 Somatization	0.63	0.63	0–4	1.59	2.14

Note: CPV = child-to-parent violence; CR = children’s report; PR = parents’ report.

**Table 2:** Spearman’s Rho correlation coefficients.

	1	2	3	4	5	6	7	8	9
1 CPV (CR)	1								
2 CPV (PR)	0.27 ***	1							
3 Self-efficacy	−0.16 *	−0.21 **	1						
4 Depression	0.06	0.26 ***	−0.32 ***	1					
5 Anxiety	−0.03	0.24 **	−0.24 **	0.61 ***	1				
6 Hostility	0.18 *	0.36 ***	−0.32 ***	0.46 ***	0.52 ***	1			
7 Obsessive–compulsive	0.14	0.27 ***	−0.28 ***	0.49 ***	0.61 ***	0.43 ***	1		
8 Interpersonal sensitivity	0.10	0.34 ***	−0.27 ***	0.66 ***	0.51 ***	0.45 ***	0.63 ***	1	
9 Somatization	−0.02	0.17 *	−0.06	0.63 ***	0.52 ***	30 ***	0.59 ***	29 ***	1

Note. *** *p* < 0.001 ** *p* < 0.01 * *p* < 0.05. CPV = child-to-parent violence; CR = children’s report; PR = parents’ report.

## Data Availability

Data are available under request.
